# Factors associated with viral suppression among adolescents on antiretroviral therapy in Free State province, South Africa

**DOI:** 10.4102/sajhivmed.v23i1.1356

**Published:** 2022-06-13

**Authors:** Balsam A.Y. Elashi, Brian E. van Wyk

**Affiliations:** 1 School of Public Health, Faculty of Community and Health Sciences, University of the Western Cape, Cape Town, South Africa

**Keywords:** HIV, viral suppression, adolescents, retention in care, antiretroviral therapy

## Abstract

**Background:**

In 2019, about 1.7 million adolescents between the ages of 10 and 19 years were living with HIV worldwide, of which 170 000 were newly infected with HIV in 2019. South Africa has the highest number of persons living with HIV. Although there has been major improvement in access to antiretroviral therapy (ART), it is still unclear what proportion of adolescents (aged 10–19 years) are virally suppressed in the provinces of South Africa.

**Objectives:**

To determine the prevalence of and the factors associated with viral suppression among adolescents (10–19 years) on ART in the Thabo Mofutsanyane District Municipality of the Free State province of South Africa.

**Method:**

A retrospective cross-sectional analysis of demographic, clinical and treatment-related information that were extracted from an electronic database was conducted using Statistical Package for the Social Sciences version 26.

**Results:**

The median duration on ART was 6.58 years. Although 78% (*n* = 4520) of adolescents living with HIV who were on ART achieved viral suppression (< 1000 copies/mL), only 9.5% (*n* = 430) were fully suppressed at < 50 copies/mL. In multivariate analysis, the odds of being virally suppressed reduced with increasing age at ART initiation. Adolescents with CD4 counts greater than 500 cells/mm^3^ at baseline had a higher odds ratio of viral suppression (adjusted odds ratio [AOR]: 1.77; confidence interval [CI]: 1.28–2.47). The odds of viral suppression were significantly lower among those not retained in care (AOR: 0.45; CI: 0.35–0.58).

**Conclusion:**

Tailored interventions should be developed to improve viral suppression among adolescents on ART.

## Introduction

Adolescents and young people represent a growing number of people living with HIV worldwide. In 2019, there were approximately 1.5 million adolescents living with HIV (ALHIV) in sub-Saharan Africa; roughly 88% of the total global number.^1^ South Africans account for a third of all new HIV infections in southern Africa, a region regarded as the epicentre of the worldwide HIV epidemic.^2^ It is estimated that there are 720 000 HIV-infected youth aged 15–24 years in South Africa.^3^ South Africa has the largest antiretroviral therapy (ART) programme globally, with 68% of people living with HIV knowing their HIV status and enrolled on ART.^4^ Nevertheless, adolescents on ART have the highest AIDS mortality rates compared to other population groups.^5^ These high adolescent mortality rates are explained by low ART adherence and high rates of attrition at all stages of the treatment cascade.^6,7,8,9^ It is reported that less than half of ALHIV in South Africa are virally suppressed.^10^ The greater likelihood of detachment from health care among ALHIV may be partly explained by their unique psychological and medical needs that are unrecognised in the health system.^11,12^ Adolescents experience a transition stage in their development and they need to be guided, mentored and counselled to adapt to the rapid psychological, biological, physical and structural changes in their lives.^5^ Moreover, young adolescents who transition to adult care, may require changes in ART dosing and in the regimen itself, which may also affect their adherence to ART.^5^ Furthermore, low adherence and retention rates can be attributed to the lack of adolescent-specific services in health care facilities, and the inadequate experience and practice of healthcare workers in dealing with ALHIV.^5,13^ Other barriers include long travelling times and distances to the health facility, the stigma of being recognised at a clinic by friends and fellow students, an elderly caregiver and high transport costs.

Despite the high frequency worldwide of suboptimal viral suppression levels among adolescents on ART, the group is often overlooked because programmatic reporting rather focuses on other groups: paediatric (0–14 years) and adult (15 years and older). Furthermore, there is an insufficient number of studies of viral load suppression (VLS) using secondary data derived from adolescents in public health settings. An analysis of the factors that affect viral suppression among ALHIV is critical to the development, at the local level, of adolescent interventions and the achievement of the final 95% goal of the Joint United Nations Programme on HIV/AIDS (UNAIDS) 95-95-95 targets.^14^

## Objectives

The objectives of the current study were to determine demographic, clinical and treatment characteristics associated with VLS among ALHIV on ART in the Thabo Mofutsanyane District Municipality of the Free State province of South Africa.

## Methods

### Study design

A retrospective, quantitative cross-sectional study was conducted to determine the factors associated with VLS among adolescents.

### Study context

In the Free State province, the Thabo Mofutsanyane District has the highest number of HIV cases (*n* = 100 361)^15^ and, with regard to incidence rates, ranks second highest in the province, with 10.6 new infections per 100 000 population.^16^ It was against this background that the aim of this study was to determine factors associated with viral suppression among adolescents on ART in this district.

### Study population and sampling

The study population consisted entirely of adolescents, aged 10–19 years, on ART and living in the Thabo Mofutsanyane District Municipality of the Free State province (*n* = 4553) in 2019. The study sample included those who were on ART for a minimum of 6 months and who had at least one documented viral load (VL) on ART in the last 12 months (*n* = 4475).

### Data collection

Routine data were extracted from an electronic database, the Three Interlinked Electronic Registers.Net (TIER.Net), after determining the population of HIV-infected adolescent patients initiated on ART from all the facilities in the study region. TIER.Net is a commonly used electronic database in public health facilities in South Africa and monitors the baseline clinical care and treatment of HIV patients.^17^ Viral load was the primary outcome variable: VL < 50 RNA copies/mL was categorised as ‘fully suppressed’; VL = 50–999 copies/mL was categorised as ‘transient suppression’; and VL > 1000 copies/mL was categorised as ‘unsuppressed’, as per the national ART clinical guidelines.^18^ Viral ‘suppression’ in this study includes all participants who had a latest VL < 1000 copies/mL recorded on the register.

Demographic variables included gender, current age and age at ART initiation. Clinical characteristics included the World Health Organization (WHO) clinical stage, the CD4 count, pregnancy, a history of tuberculosis and the use of Isoniazid Preventive Therapy and Cotrimoxazole, all at baseline (at ART initiation). The duration of ART was included. Information on retention in care was used to distinguish between those in care; those registered as dead, transferred out and those lost to follow-up, as per national ART guidelines.^18^

### Data analysis

Extracted data were entered into an Excel spreadsheet, cleaned and thereafter imported into Statistical Package for the Social Sciences (SPSS) version 26 (2019, IBM Corp, Armonk, New York, United States [US]) for analysis. Descriptive frequency tables were created for categorical variables outlining the number of adolescents in the various categories and the proportions, using percentages. Continuous data were also presented either as means with standard deviations or as medians with interquartile ranges (IQR). Bivariate analysis was used to determine the significance and strength of associations between VLS, at < 1000 copies RNA/mL, and the exposure variables as indicated above. Statistical significance was tested using the chi-square test with significance set at *P* < 0.05. Multivariate logistic regression identified factors independently associated with viral suppression and presented as adjusted odds ratios (AOR) with 95% confidence intervals (95% CI).

## Results

The median age of adolescents in this study was 15 years (IQR: 13–18 years). Most were between 15 and 19 years (68.2%; *n* = 3083) and were female (56.2%; *n* = 2539). The median age at ART initiation in this cohort was 8 years (IQR: 5–12), and the median CD4 count at ART initiation was 285 cells/mm^3^ (IQR: 103–490). The median duration of treatment was 6.6 years (IQR: 3.58–8.75).

Approximately half were initiated on Abacavir/Lamivudine/Efavirenz (A3E) (50.7%; *n* = 2252); followed by Stavudine/Lamivudine/Efavirenz (S3E) (19.0%; *n* = 845) and Tenofovir/Emtricitabine/Efavirenz (TFE) (14.5%; *n* = 643). At the time of data extraction, 46.1% (*n* = 2084) were on A3E, with 7.5% (*n* = 340) on Abacavir/Lamivudine/Lopinavir/ritonavir (A3L), and 23.7% (*n* = 1070) on TFE.

At the time of the study, 70.1% (*n* = 3167) were in care; while 16.3% (*n* = 736) had transferred, 11.6% (*n* = 523) were lost to follow-up and 2.1% (*n* = 94) had died.

Most participants (68.7%; *n* = 3107) were categorised as transiently suppressed (50–999 RNA copies/mL); while only 9.5% (*n* = 430) were fully suppressed (< 50 RNA copies/mL) and 21.8% (*n* = 983) were unsuppressed (≥ 1000 RNA copies/mL).

In bivariate analysis, viral suppression was significantly associated with current age (*P* = 0.006) and age at ART initiation (*P* < 0.001) (Table 1). There were significant associations between viral suppression and adolescents’ baseline CD4 count and WHO stage. Viral suppression was greater in those with CD4 counts > 500 cells/mm^3^ (83.93%; *n* = 653). World Health Organization stage IV had the highest viral suppression (81.20%; *n* = 95), followed by WHO stage I (80.57%; *n* = 1617).

**TABLE 1:** Viral load by demographic and clinical characteristics of adolescents on ART in the Thabo Mofutsanyane District, Free State province, 2019.

Categories	Viral load (in copies RNA/mL)				*P*
	< 1000		≥ 1000		
	*n*	%	*n*	%	
Total	3537	78.25	938	21.75	
**Current age (in years)**					0.006
10–14	1160	80.72	277	19.28	
15–19	2377	77.10	706	22.90	
**Gender**					0.079
Male	1526	77.03	455	22.97	
Female	2011	79.20	528	20.80	
**Age at ART initiation (in years)**					< 0.001*
0–4	839	83.48	166	16.52	
5–9	1375	76.22	429	23.78	
10–14	876	74.87	294	25.13	
15–19	447	82.62	94	17.38	
**Baseline CD4 count (cells/mm^3^)**					< 0.001*
< 200	843	72.11	326	27.89	
200–500	1014	78.12	284	21.88	
>500	653	83.93	125	16.07	
**WHO stage**					0.001*
I	1617	80.57	390	19.43	
II	591	74.43	203	25.57	
II	497	75.30	163	24.70	
IV	95	81.20	22	18.80	
**History of tuberculosis**					0.003*
Yes	228	71.25	92	28.75	
No	2121	78.50	581	21.50	
**Pregnant, females (*N* = 2111)**					0.980
Yes	125	79.11	33	20.89	
No	1986	79.19	522	20.81	
**Received IPT**					0.680
Yes	717	79.14	189	20.86	
No	1992	78.49	546	21.51	
**Cotrimoxazole**					0.150
Yes	1206	76.77	365	23.23	
No	1768	78.72	478	21.28	
**Duration on ART (in years)**					0.045*
< 1	135	85.44	23	14.56	
1–2	297	80.05	74	19.95	
3–5	891	78.85	239	21.15	
6–10	1728	76.73	524	23.27	
> 10	486	79.80	123	20.20	
**Retention in care**					< 0.001*
In care	2579	81.43	588	18.57	
Lost to follow-up/died/transferred	958	70.81	395	29.19	

*N* = 4475.

IPT, isoniazid preventive therapy; WHO, World Health Organization.

* , denotes statistical significance at *P*< 0.05.

Furthermore, clinical factors such as history of tuberculosis (*P* = 0.003), duration on ART (*P* = 0.045), ART regimen at baseline (*P* < 0.001) and current ART regimen (*P* < 0.001) were also significantly associated with viral suppression.

Adolescents who were retained in care had higher viral suppression rates compared to those who were not retained (81.43% vs 70.81%; *P* = < 0.001).

In Table 2, the results of the multivariate logistic regression analysis are shown: age at ART initiation, CD4 count at baseline, baseline ART regimen, current ART regimen and retention in care were significantly associated with viral suppression. Reduced odds of being virally suppressed were associated with higher age at ART initiation. When comparing other age groups to ART initiation at 0–4 years, it is evident that those who started ART between 5 and 9 years (AOR = 0.52; confidence interval [CI]: 0.34–0.80), between 10 and 14 years (AOR = 0.41; CI: 0.23–0.72) and between 15 and 19 years (AOR = 0.39; CI: 0.17–0.93) were less likely to be virally suppressed.

**TABLE 2:** Determinants of viral non-suppression (≥1000 RNA copies/mL) among adolescents on ART in the Thabo Mofutsanyane District, Free State province, 2019 (*N* = 4520).

Categories	Adjusted OR	95% CI
**Age (in years)**		
10–14	1	-
15–19	1.32	0.93–1.88
**Gender**		
Male	1	-
Female	1.09	0.86–1.37
**Age at ART initiation (in years)**		
0–4	1	-
5–9	0.52	0.34–0.80
10–14	0.41	0.23–0.72
15–19	0.39	0.17–0.93
**Baseline CD4 count (cells/mm^3^)**		
< 200	1	-
200–500	1.24	0.96–1.62
> 500	1.77	1.28–2.47
**WHO stage**		
I	1	-
II	0.78	0.59–1.03
III	0.92	0.68–1.26
IV	1.75	0.81–3.78
**History of tuberculosis**		
No	1	-
Yes	0.78	0.50–1.02
**Duration on ART (in years)**		
< 1	1	-
1–2	1.07	0.39–2.92
3–5	1.06	0.41–2.75
6–10	1.03	0.38–2.79
> 10	0.86	0.28–2.62
**Retention in care**		
In care	1	-
Lost to follow-up/died/transferred out	0.45	0.35–0.58

Note: Bold data shows significance at 5% significance level.

OR, odds ratio; 95% CI, 95% confidence interval; WHO, World Health Organization; ART, antiretroviral therapy.

A higher CD4 count at baseline was associated with higher odds of viral suppression, and adolescents who had a CD4 count > 500 cells/mm^3^ (AOR = 0.77; CI: 1.28–2.47) were 77% more likely to be virally suppressed compared to adolescents who had a CD4 count < 200 cells/mm^3^.

## Discussion

Our findings indicate that the progression of adolescents on ART in the Thabo Mofutsanyane District Municipality of the Free State province to the final ‘95’ of viral suppression in the UNAIDS 95-95-95 treatment trajectory, remains low at 78%. Our finding is lower than that reported in a meta-analysis by Zanoni et al., of South African adolescents and young adults on ART, where 81% were virally suppressed (< 1000 copies RNA/mL).^3^ However, in the Eastern Cape and Mpumalanga, in settings reflecting conditions similar to those reported in the current paper, 71% of adolescents in the Eastern Cape and 74% of those in Mpumalanga were virally suppressed in the past 12 months.^19,20^ Our study extends the evidence in support of an optimised adolescent HIV care cascade for all South African ALHIV across all healthcare facilities.^21^

Our study confirms the association between baseline CD4 count levels and viral suppression. We found that adolescents in our analysis follow the pattern reported elsewhere, where people commencing ART with less advanced immunodeficiency (CD4 > 200 cells/mm^3^ – 350 cells/mm^3^) appear to have better virological outcomes than those who commence with more severe immunodeficiency (CD4 count < 200 cell/mm³).^22,23,24,25^ However, other studies found that higher CD4 counts were not associated with viral suppression.^26,27,28^ Thus, further qualitative research is needed to explain the association of initiating ART when symptomatic (which is generally associated with low CD4 counts at baseline) and subsequent health behaviours (adherence and engagement in care) impact viral suppression.

We found that adolescents who initiated ART at an older age were less likely to be virally suppressed. This suggests that those on ART for a long time are more ‘settled’ in care and express this by being virally suppressed. Children starting ART at a younger age may be more accustomed to taking medication and more settled in a daily routine. These children may develop better adherence patterns as they transition to adolescence, provided that stable support is given.^29^

Prolonged retention in care is associated with optimal adherence and the reduced likelihood of high VL in patients on ART.^30^ In the current study, retention in care was significantly associated with viral suppression as confirmed in other studies.^30,31^ Widening the scope of an HIV programme to specifically target youth (15–24 years of age), with sexual and reproductive health services and support groups in addition to ART, has been reported by Lamb et al. to significantly lower attrition rates.^32^ Moreover, Massavon et al. reported that higher retention in care rates are achieved in community home-based care services (94.0%) than in facility-based/family-centred care services (84.7%).^33^

### Limitations

An important limitation of this study has been missing information from routine records. This may have affected the analysis of variables associated with viral suppression. The study also involved retrospective extraction of data from a database, restricting us to routinely collected variables. This limited the extent to which other variables could also be explored, such as social, cultural and economic factors that can also affect viral suppression. Specifically, it is noted that there may be considerable differences in adolescents that are infected perinatally versus behaviourally. Unfortunately, this point was not part of our routine data and we were not able to test for a difference. A standard VL result of 124 copies/mL is captured in TIER.Net for persons with a laboratory result reported as ‘lower than the detectable level’, rather than as an actual value.

Cross-sectional studies do not allow causality to be established and, in particular, with regard to VL testing, these events were not co-incident with several co-variates recorded in the study, for example, the WHO stages and the occurrence of tuberculosis. The significance of such factors in relation to viral suppression could not reliably be determined in this study. Further, we note the limitation that retention in care status was not assessed at the same time as VLS. Therefore, the significant association between retention in care and VLS should be interpreted with caution. Notwithstanding, there are several studies that support this association – therefore this result is likely to be valid.

## Conclusion

Viral suppression of adolescents on ART in the Thabo Mofutsanyane District at 78% is below the target of 95% set by UNAIDS, and there is an urgent need for interventions to improve individual behaviour, such as the earlier initiation on ART and retention in care, that would lead to improved rates of viral suppression. It is further disconcerting that when considering the higher threshold of viral load < 50 copies/mL as fully suppressed, only 9.5% achieved this rate. This finding does not bode well for achieving global efforts to reach 95-95-95 results by 2030, as propagated by UNAIDS.^14^ Routine monitoring of viral load results should be enhanced to detect possible VL failure earlier, so that timely interventions can be put in place. Further qualitative research is recommended to explore explanations for failure to achieve viral suppression.
